# Adsorbing/dissolving Lyoprotectant Matrix Technology for Non-cryogenic Storage of Archival Human Sera

**DOI:** 10.1038/srep24186

**Published:** 2016-04-12

**Authors:** Morwena J. Solivio, Rebekah Less, Mathew L. Rynes, Marcus Kramer, Alptekin Aksan

**Affiliations:** 1 Biostabilization Laboratory, Department of Mechanical Engineering, University of Minnesota, Minneapolis, MN 55455, USA; 2 School of Biomedical Engineering and Sciences, Virginia Tech, Blacksburg, VA 24061, USA

## Abstract

Despite abundant research conducted on cancer biomarker discovery and validation, to date, less than two-dozen biomarkers have been approved by the FDA for clinical use. One main reason is attributed to inadvertent use of low quality biospecimens in biomarker research. Most proteinaceous biomarkers are extremely susceptible to pre-analytical factors such as collection, processing, and storage. For example, cryogenic storage imposes very harsh chemical, physical, and mechanical stresses on biospecimens, significantly compromising sample quality. In this communication, we report the development of an electrospun lyoprotectant matrix and isothermal vitrification methodology for non-cryogenic stabilization and storage of liquid biospecimens. The lyoprotectant matrix was mainly composed of trehalose and dextran (and various low concentration excipients targeting different mechanisms of damage), and it was engineered to minimize heterogeneity during vitrification. The technology was validated using five biomarkers; LDH, CRP, PSA, MMP-7, and C3a. Complete recovery of LDH, CRP, and PSA levels was achieved post-rehydration while more than 90% recovery was accomplished for MMP-7 and C3a, showing promise for isothermal vitrification as a safe, efficient, and low-cost alternative to cryogenic storage.

Cancer is one of the leading causes of mortality, accounting for approximately 23% of all deaths in the U.S. each year^1^. Early detection and continuous monitoring for recurrence are essential for a positive prognosis, as it is at its initial stages that the disease is most responsive to therapeutic intervention. Early detection focuses on diagnosing the disease before clinical symptoms arise; for example, by detecting the presence of certain cancer biomarkers found in bodily fluids such as the blood^2^,^3^. Therefore, studies focusing on discovery of highly sensitive and specific cancer biomarkers have become increasingly prevalent^3^,^4^,^5^.

In spite of the advances in fast and sensitive analytical detection methodology and the vast amount of research conducted evaluating thousands of molecular signatures as potential biomarkers for cancer (detailed in more than 150,000 reports published to date), less than two dozen biomolecules have currently been approved for clinical use by the Food and Drug Administration (FDA)^6^,^7^. An even smaller number is found in the blood, which is home to more than 10,000 potential biomarkers^8^,^9^. One of the main reasons for the inefficient and slow progress is the poor informational quality of the collected human biospecimens (tissue samples, bodily fluids, etc.) used in biomarker detection and validation studies. A significant fraction of the collected biospecimens is known to be compromised due to sub-optimal handling and storage conditions^10^,^11^.

Biomarker development is composed of a series of phases including discovery, verification, and clinical validation, which require large numbers of high quality biospecimens^12^. For this purpose, millions of  “archival” biospecimens are continuously being collected and stored in biorepositories and biobanks across the world^13^ following standardized collection, handling, and storage protocols to minimize “pre-analytical variability”^14^,^15^. Cryogenic storage (at −20 °C, −80 °C, and in liquid nitrogen) is the most ubiquitous method for preserving liquid biospecimens^16^. However, even when best practices are followed and the biospecimens are immediately frozen after collection and processing, freeze/thaw processes alone can damage proteinaceous biomarkers by mechanisms such as cold denaturation^17^, binding and aggregation at the ice interface^18^,^19^, degradation due to ionic gradients and pH swings^20^, physical separation of the lyo-/cryoprotectant^21^, dissociation^22^, and oxidative damage^23^.

Certain serum biomarkers are known to be especially vulnerable to cryogenic storage. Lactate dehydrogenase (LDH), a biomarker currently being evaluated for various types of cancer is known to be sensitive to the presence of an ice interface and thus is unstable during cryogenic storage and freeze-thaw^24^,^25^. Similarly, the level of C3a, one of the proteins formed by the cleavage of complement component 3 (C3) and a breast cancer biomarker, is known to increase by more than 50% during extended cryogenic storage and freeze-thaw^26^. The matrix metalloproteinase (MMP) family (specifically, MMP-1, MMP-7, MMP-9, and MMP-13), identified as diagnostic and prognostic biomarkers in breast, lung, and pancreatic cancers is also shown to be highly susceptible to freeze-thaw damage^27^,^28^. In addition to damage imposed by cryogenic storage on numerous proteinaceous biomarkers, frozen state storage is extremely costly (requiring large, dedicated, well-controlled, and equipped spaces with very large carbon footprint)^29^. Storage of the existing +600 million biospecimens (increasing at a rate of 20 million samples/year) is assumed to cost $100 million annually^16^,^29^,^30^.

Room temperature stabilization and storage emerges as a viable alternative to cryogenic storage. Isothermal vitrification involves desiccation of liquids containing high concentrations of glass-forming lyoprotectants (carbohydrates such as trehalose, sucrose, hydroxyethyl starch, and dextran), under non-cryogenic conditions into a very viscous fluid (a glass)^31^. The glassy state is characterized by exceedingly low molecular mobility, which inhibits biochemical reactions and deterioration of biospecimens, preserving the macromolecules in their native states^32^,^33^,^34^,^35^. This strategy is utilized in nature by desiccation-resistant species to transition into a state of dormancy (anhydrobiosis) when water is scarce^36^. Many anhydrobiotic organisms (such as tardigrades, lichen, some ferns, seeds, yeast, and bacteria) accumulate large amounts of carbohydrates such as sucrose or trehalose to achieve this feat^36^.

In this communication, we present a novel carbohydrate-based adsorbing/dissolving lyoprotectant matrix to stabilize a broad range of serum-based proteinaceous cancer biomarkers at non-cryogenic temperatures. Trehalose, a well-known cryo-/lyoprotectant is used as one of the main excipients, along with the polysaccharide dextran, utilized for its high glass transition temperature (T_g_)^31^. The lyoprotectant matrix is engineered to rapidly adsorb a liquid biospecimen (e.g. human serum or plasma) while dissolving in and uniformly mixing with it. The biospecimen is then isothermally desiccated, enabling it to transition into a glass. At the glassy state, the biomarkers in the biospecimen are stabilized and the sample can be stored long term at non-cryogenic conditions. As the matrix is completely water soluble, total protein recovery post-storage is achieved, circumventing a significant problem inherent to blot-based preservation methods^37^. Note that there are currently very few commercially available room temperature stabilization products (only for DNA and RNA)^31^, but no technology is available for the stabilization of a large spectrum of serum biomarkers in the dried state.

## Results and Discussion

To effectively stabilize biological samples by isothermal vitrification, high concentrations of lyoprotectants are required. However, it is extremely difficult to uniformly mix carbohydrate-based lyoprotectants at concentrations of 1–2 M with liquid biospecimens of high protein content (such as serum)^35^, as the sugars tend to form undissolved aggregates (clumps), requiring rigorous mixing, a process that is detrimental to serum proteins. Non-uniform mixing of sugars and heterogeneity introduced during drying, on the other hand, are some of the main reasons for sample degradation during dried/vitrified state biopreservation^35^,^38^. The adsorbent/dissolving matrix technology developed here is designed to bypass these problems while enabling manufacturing scalability.

**Figure 1:**
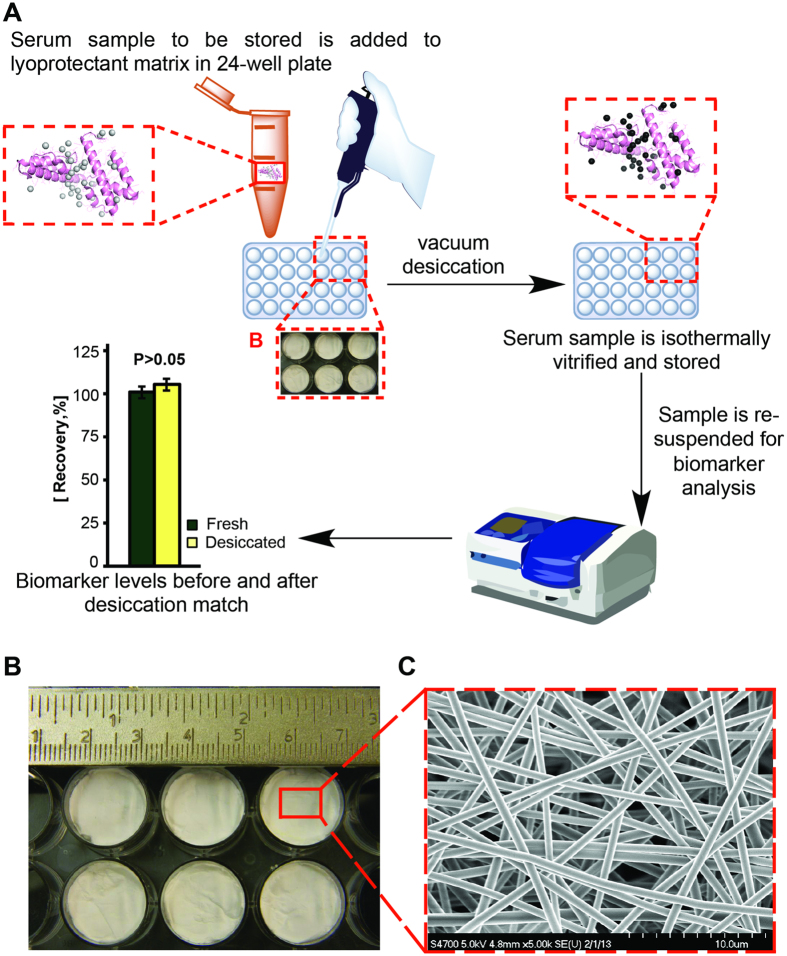
Isothermal vitrification methodology and matrix. (**A**) Schematic for sample stabilization methodology: 150 μL of serum sample to be stored is added to 250±5 mg lyoprotectant matrix in 24-well plate. Before desiccation, the protein biomarker in serum (inset) hydrogen bonds with water molecules (light gray spheres). The sample is desiccated in vacuum where the water molecules are replaced with excipient molecules (inset; black spheres). Isothermal vitrification raises sample T_g_ allowing for storage at RT. Sample is re-hydrated for biomarker analysis. The optimal result is the preservation of the biomarker such that comparable level before and after desiccation is achieved (P>0.05). The protein image (inset) was obtained from the RCSB PDB (www.rcsb.org), PDB ID 4HW5 (G. Bajic, L. Yatime, A. Klos, G.R. Andersen (2013) structure of Human C3a Protein Sci 22: 204–212) and the figure generated with Pymol (DeLano, W. L. (2002) The PyMOL Molecular Graphics System. DeLano Scientific)^65^,^66^ (**B**) The matrix packaged in a 24-well plate, ready for use (**C**) SEM image of the electrospun adsorbing/dissolving matrix.

A schematic of the anticipated procedure for sample stabilization using the optimized isothermal vitrification matrix and method developed here is shown in Fig. 1A. The archival serum samples to be stored are transferred to 24-well plates (150 μL serum/well), which are pre-packed with approximately 250 mg of the adsorbent matrix/well (Fig. 1A,B). Thanks to the capillary forces induced by the porous nature of the electrospun lyoprotectant matrix (Fig. 1C), the serum is uniformly adsorbed in, while the matrix slowly dissolves and thoroughly mixes with the serum (without forming any clumps, skin, etc.). The sample is then vacuum dried, during which, lyoprotectants (Fig. 1A, inset, black spheres) stabilize the protein structure, mainly by replacing water (Fig. 1A, inset, light gray spheres)^39^,^40^. The desiccated samples are then stored until use. The goal is to reach a high level of stability such that biomarker levels in stored samples match those in fresh samples within 5% error.

### Electrospinning

**Figure 2:**
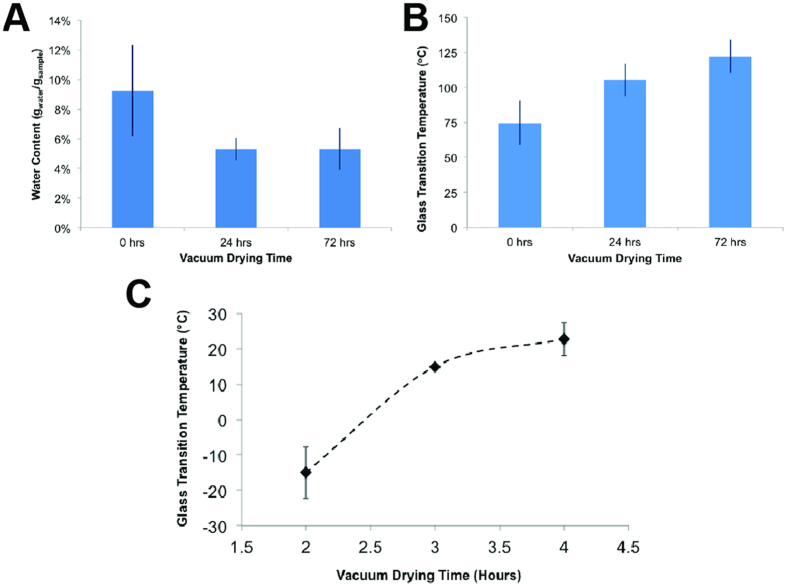
Matrix characterization. (**A**) Water content of the electrospun matrix with vacuum drying. Water content before vacuum drying is 9.69 ± 0.65%. Drying for 24 hours reduced water content to 5.59 ± 0.43%, while water content after 72 hours of drying is 5.30 ± 0.01% (P = 0.5; 24 and 72 hours). (**B**) The corresponding change in the glass transition temperature (T_g_) of the electrospun matrix with vacuum drying. Matrix T_g_ before drying is 75 ± 16 °C. Drying for 24 hours increased T_g_ to 105 ± 12 °C, while 72 hours of drying resulted to a T_g_ of 122 °C ± 12 °C (P = 0.46; 24 and 72 hours) (**C**) T_g_ of the human serum adsorbed into the matrix and vacuum dried. Sample T_g_ after 2 hours of drying is −14 ± 10 °C. Drying for 3 hours significantly increased T_g_ to 15 ± 0 °C (P = 0.0037; 2 and 3 hours). Sample T_g_ further increased to 23 °C ± 5 °C after 4 hours of drying (P = 0.025; 3 and 4 hours). Error bars represent standard deviations from at least 3 independent experiments.

A high voltage differential was utilized to extrude the viscous lyoprotectant cocktail to form fibers of 4–5 μm in diameter (Fig. 1C). Multiple layers of fibers were woven together and dried to produce a highly adsorbent and porous matrix (the sponge) (Fig. 1B). Electrospun matrices were compacted in 24-well plates (250 ± 5 mg/well) and vacuum dried for 24 hours to reduce their water content, which in turn, enhanced the T_g_. Before vacuum drying, the water content of the matrix was 9.69 ± 0.65% (w/w), which decreased to 5.59 ± 0.43% after 24 hours of drying (Fig. 2A). Drying the matrix beyond 24 hours did not significantly alter its water content (5.30 ± 0.01% at 72 hours; P = 0.5), while very slightly increasing its glass transition temperature, although the increase was not statistically significant (T_g_ = 105 ± 12 °C with 24 hours of drying, and T_g_ = 122 ± 12 °C with 72 hours of drying; P = 0.46) (Fig. 2B).

The human serum samples (or the model serum solution) used in the experiments outlined here were then transferred to 24-well plates (150 μL serum/well) containing the adsorbent matrix (Fig. 1B). As expected, T_g_ increased with increasing sample drying period (Fig. 2C), with −14 ± 10 °C, 15 ± 0 °C, and 23 ± 5 °C after 2, 3, and 4 hours of drying, respectively (P = 0.025; 3 and 4 hours drying). Giving a high T_g_ at a reasonably short time, 4 hours was selected as the desiccation period to be used in this work, at the end of which, the sample was ready for storage in a standard refrigerator as its glass transition temperature (23 ± 5 °C) already exceeded 4 °C. The 24-well plates were stored in the dark, in a dry environment. At the end of the storage period, the vitrified samples were re-hydrated by adding 100 μL distilled water (volume lost during drying) and 1.35 mL 1X PBS (total re-suspension volume is ten times the original volume), and analytical testing was conducted.

### High Abundance Protein Response to Desiccation: Experiments with Human Serum

**Figure 3:**
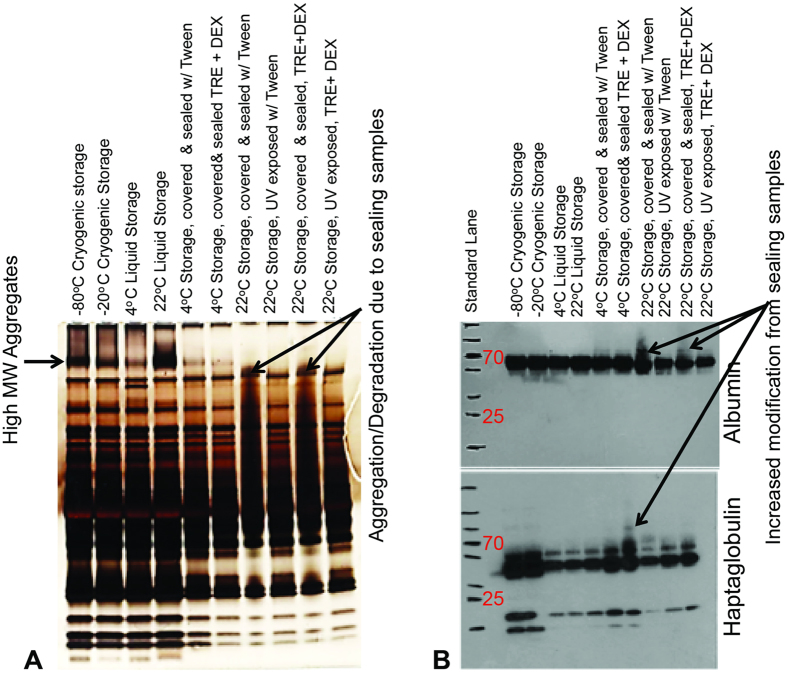
Sample experimental result from Table 1. Human serum samples isothermally vitrified and stored for two weeks at 4 °C vs. 22 °C in sealed (constant relative humidity) vs. unsealed (uncontrolled relative humidity) conditions are compared to samples stored at −80 °C, −20 °C, 4 °C, and 22 °C without any cryoprotectant. (**A**) SDS-PAGE and silver staining were used to determine degradation and aggregation behaviors of total serum proteins exposed to various storage conditions. The serum proteins were prone to aggregation when stored in frozen and liquid states (lanes 1–4) or when vitrified, sealed, and stored at 4 °C (lanes 5–6). Sealing of vitrified samples and storage at 22 °C promotes both aggregation and degradation (lanes 7 and 9). Isothermally vitrified samples did not exhibit aggregation nor degradation when stored un-sealed (lanes 8 and 10). (**B**) Western Blot analysis was used to monitor modifications to the HAPs, albumin and haptaglobulin following storage. Albumin and haptaglobulin were unaffected by liquid and frozen storage. Sealed storage in the vitrified state resulted to modification on both proteins only when Tween was included in the matrix (lane 7).

At the early stages of isothermal vitrification matrix development, a significant number of short and medium term stability experiments were conducted with human serum isolated from whole blood (as detailed in Materials and Methods section) to determine the stability (aggregation and degradation) of selected high abundance proteins (HAP), albumin and haptaglobulin against desiccation. The details of the experiments conducted and the results are presented in Table 1. In these experiments, the changes in HAP signatures in serum samples isothermally vitrified using different versions of the lyoprotectant matrix (V1, V2, and V3) were compared to those of frozen samples stored at −20, −40, or −80 °C, and also to those exposed to repeated freeze/thaw. Matrix V1 contained only trehalose and dextran. Matrix V2 included 0.5% Tween 20, in addition to trehalose and dextran. Matrix V3 included 0.5% Tween 20 and 3% glycerol, on top of trehalose and dextran. In preventing HAP aggregation during desiccation, Tween 20 and glycerol had minimum effect, but it was noted that following isothermal vitrification and re-hydration, aggregation of HAPs was not worse than that observed after freeze/thaw. A sample result obtained from the experiments outlined in Table 1 is presented in Fig. 3. In this particular experiment, 4 °C or 22 °C storage (in sealed containers with controlled humidity vs. unsealed containers) for two weeks after isothermal vitrification was compared to frozen state storage at −20 °C and −80 °C, and liquid state storage at 22 °C and 4 °C. Figure 3A shows a sample silver stained gel profile for serum samples stored at −80 °C, −20 °C, 4 °C, and 22 °C (lanes 1–4 from the left), and isothermally vitrified samples stored at various conditions (lanes 5–10). Note that the high molecular weight aggregates (marked by an arrow at the top of the gel image) are more pronounced in samples kept in liquid and cryogenic storage: the highest level observed in samples kept at room temperature (22 °C), followed by samples stored at −80 °C, −20 °C, and are at the lowest level in samples stored at 4 °C. These aggregates were also observed at much lower levels in isothermally vitrified samples (in matrices with and without Tween 20) stored at 4 °C that were covered and sealed (note the faint bands on lanes 5–6 aligned with those marked aggregates in frozen and liquid state stored samples). Samples that were covered and sealed showed both aggregation, and degradation behavior if kept at 22 °C (note vertical streaking on lanes 7 and 9). This was not observed in the unsealed samples (lanes 8 and 10). UV exposure of desiccated samples did not increase aggregation or degradation. In a separate experiment, we determined that unsealed, isothermally vitrified samples, with and without Tween 20, stored at 22 °C showed a different profile, with the absence of the high molecular weight aggregates observed in samples kept in cryogenic conditions (Figure S1), which indicated that desiccated storage in unsealed containers did not promote as much aggregation as cryogenic state storage. Sealing during desiccation caused proteins to aggregate, which was exacerbated by the presence of Tween 20. The presence of Tween 20 alone in the matrix however, did not cause aggregation.

Figure 3B shows a typical Western Blot analysis conducted on HAPs following storage. No albumin aggregation/modification was observed in samples stored in frozen and liquid state (lanes 1–4), indicating that the high molecular weight bands observed in the silver stained gels were not due to alterations in albumin, while albumin modification likely contributed to the bands observed in sealed, isothermally vitrified samples. The observations for silver staining of total serum protein coincided well with Western Blot results for albumin in isothermally vitrified samples. The highest level of albumin modification was observed for sealed samples containing Tween 20, stored at 22 °C (note vertical streaking on lane 7). The presence of Tween 20 alone however, did not result in any detectable change, while sealing alone resulted in modification. These observations indicated that sealing promoted changes to albumin, which was intensified in the presence of Tween 20. A similar profile was observed for haptaglobulin throughout all samples, except for the sample containing Tween 20, which was sealed and stored at 22 °C (lane 7). We find that for serum samples that were desiccated and stored at 4 °C and 22 °C, with or without Tween 20 in unsealed containers, the level of protein modification is minimal when compared to samples stored cryogenically.

Stabilization of high abundance serum proteins is important for overall biomarker preservation, as these proteins likely interact with the low abundance biomarkers that are present in the serum. For instance, certain proteins exhibit higher storage stability in the presence of high concentrations of serum albumin^41^,^42^. If high abundance biomarkers aggregate or degrade, their concentration in the serum declines, and their stabilizing effects likely diminish^41^. Additionally, high abundance biomarkers can act as carrier proteins for important low molecular weight (LMW) biomarkers, which are rapidly cleared from the blood via kidney filtration or liver uptake due to their small size. Hitching on larger, high abundance resident proteins such as albumin, serum half-lives of LMW biomarkers can significantly increase, facilitating detection^43^.

Stabilization of high abundance proteins can be readily translated to the stabilization of low abundance, LMW biomarkers. The results of the experiments outlined above were used to finalize the composition of the main lyoprotectant cocktail for the production of the electrospun isothermal vitrification matrix developed in succeeding studies (outlined below), with the main goal of stabilizing low abundance proteins (LAP) in the serum.

### Low Abundance Biomarker Response to Desiccation: Excipient Optimization

In these experiments, we aimed to enhance the desiccation stability of selected low abundance biomarkers by introducing additional protective excipients to the lyoprotectant matrix. The experiments conducted here utilized a model serum solution containing 1X PBS and 50 mg/mL bovine serum albumin (BSA) to simplify processing and increase experimental output. Post-desiccation recovery of 5 selected proteinaceous biomarkers as determined by enzyme activity assay or ELISA following desiccation/rehydration was used to optimize the low-concentration additives to be included in the matrix. The biomarkers that were selected for this purpose were; lactate dehydrogenase (LDH) (unstable against freezing and freeze/thaw)^44^, C-reactive protein (CRP) (stable against freezing, freeze/thaw, room temperature storage, and 4 °C refrigeration)^45^,^46^, total prostate-specific antigen (total PSA) (stable against freezing, freeze/thaw, and 4 °C refrigeration)^47^,^48^, matrix metalloproteinase-7 (MMP-7) (unstable against freezing and freeze/thaw)^49^, and C3a (stable against freezing but unstable against freeze/thaw and 4 °C refrigeration)^26^,^50^, representing various levels of sensitivity to different storage conditions.

The concentrations of the biomarkers spiked into the model serum solution were: 8 μg/mL for LDH (a concentration determined to be the most vulnerable to repeated freeze/thaw, allowing us to monitor changes in activity during processing), 4.0 ng/mL for PSA, 1 μg/mL for C-reactive protein, 11.5 ng/mL for MMP-7, and 11 μg/mL for C3a. These were the lower physiological threshold concentrations for these biomarkers in human serum.

### Low Concentration Excipient Selection and Formulation Optimization for LDH Stabilization

For optimization of the chemical composition of the adsorbing/dissolving lyoprotectant matrix, LDH was selected as the model protein due to its high sensitivity to processing parameters and stresses such as freeze-thaw^44^,^51^. LDH is also composed of four sub-units (a tetramer), making it a good model for highly sensitive multimeric proteins^52^. Additionally, LDH enzymatic assays are commercially available, which allows for facile monitoring of its activity.

**Figure 4:**
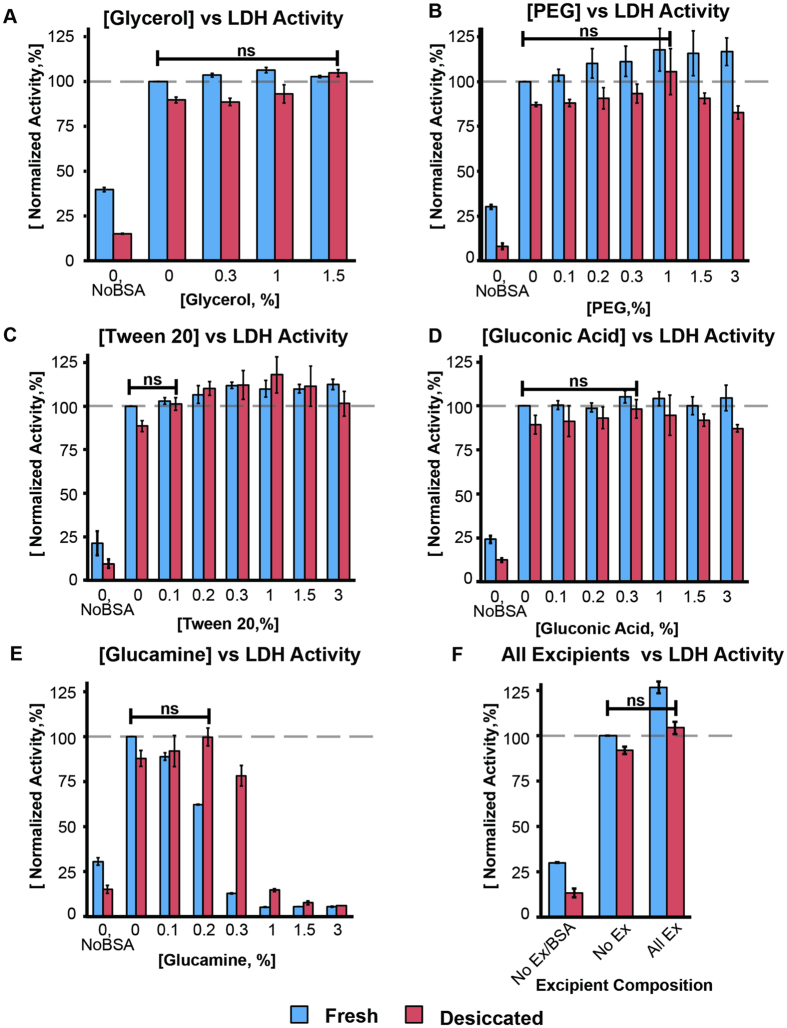
Optimization of the lyoprotectant matrix. Matrix optimization by monitoring LDH response to isothermal vitrification at the following conditions: Without any excipient, with each individual excipient at various concentrations, and with all excipients present at the determined optimum concentrations. All reported values are relative to the fresh control (without added excipients) set at 100%. Error bars represent standard deviation from at least three independent experiments. Connecting bars show excipient concentrations (**A–E**) that result in post-rehydration recovery that is not significantly (**ns**) different from the fresh, un-treated control. The lowest excipient concentration that provides the highest stability was used in the final matrix (**F**).

Figure 4A–F demonstrate the effect of various low concentration excipients on LDH activity in the model serum solution before and after desiccation. To illustrate the effect of HAPs on LAP stabilization, we measured fresh and desiccated LDH activity in the absence of BSA and compared it to the fresh control containing 50 mg/mL BSA. Fresh samples in PBS (without BSA) exhibited a significantly lower activity (29 ± 5%) compared to the control, while desiccation of these samples in matrix V1 (containing only trehalose and dextran; as described in Table 1) further reduced enzymatic activity to 12 ± 2%. For samples without BSA, the values reported are the average of the measured values in Fig. 4A–F. The ANOVA p-values were 0.15 and 0.19 for fresh and desiccated samples, respectively, indicating insignificant variations between experiments. When matrix V1 was used to stabilize the model serum solution spiked with LDH, 89 ± 2% of the original enzymatic activity was preserved post-rehydration. BSA has been shown to stabilize multimeric enzymes including LDH from freezing-induced dissociation via preferential exclusion (LDH in the presence of BSA prefers the more compact multimeric form over the dissociated monomers)^53^. The considerably higher recovery in desiccated samples containing BSA compared to those lacking BSA (89 ± 2% vs. 12 ± 2%) indicated that BSA provided protection during desiccation and rehydration in a similar manner as it does during freezing.

To further enhance LDH stability during desiccation, additional low concentration excipients targeting specific damage mechanisms were identified and used as follows:

Glycerol is a non-toxic natural osmolyte and cryoprotectant, which enhances protein stability and prevents aggregation^54^. Glycerol is also shown to be an effective protein stabilizer at the glassy state, acting synergistically with trehalose in suppressing the fast vibrations, which can result in protein denaturation and deactivation^55^.

The results for the desiccation experiments conducted with glycerol in the range of 0.3–1.5% (v/v) added to matrix V1 are shown in Fig. 4A. Enzymatic activity in fresh samples containing the test concentrations of glycerol did not deviate considerably from the control containing no excipient. In desiccated samples, LDH activity generally increased with increasing glycerol concentration, with the maximum activity achieved at 1.5% glycerol with 105 ± 2% recovery (P = 0.13). 1.5% glycerol was therefore selected to be used in the optimized matrix. Note that 10% (v/v) glycerol was shown in previous work from our group to cause HAP aggregation in human serum while decreasing the T_g_^37^.

Polyethylene glycol (PEG) is a water-soluble, biocompatible polymer shown to stabilize serum albumin^56^. PEG also protects multimeric proteins such as LDH and phosphofructokinase^53^,^57^. PEG at different molecular weights (400–20,000 Da) were tested in desiccation experiments and it was determined that 8000 Da PEG induced maximum stabilization. Concentrations of 8000 Da PEG ranging from 0.1 to 3% (w/v) were tested for LDH stabilization (Fig. 4B). LDH activity in PEG-containing fresh samples increased in proportion to excipient concentration, starting to plateau in the 1–3% PEG range with an average activity of 117 ± 1% (ANOVA P = 0.98). The 17% rise in activity compared to the untreated control was likely due to crowding, which promoted formation of the more active LDH tetramer (rather than the the dimeric form, which is abundant in serum but is known to exhibit lower activity)^58^,^59^. Increase in LDH activity is also observed after freeze/thaw (by up to 46% after ten freeze/thaw cycles; (Figure S2).

A general increase in activity was observed in desiccated samples, although the increase is minute in the 0.1–0.3% PEG range (ANOVA P = 0.15). Maximum activity was achieved at 1% PEG (106 ± 13%, P = 0.38), while the activity started to decrease at higher excipient concentrations. PEG is recognized as one of the most effective cryoprotectants, and is shown to induce stability at concentrations of 1% or less^60^, a characteristic that was observed in our experiments as well.

Tween 20 is a biocompatible surfactant that is often added to protein formulations in order to prevent damage during purification, transportation, freeze/drying and spray-drying^61^. Tween 20 acts by impeding surface or air-interface adsorption, which can result in protein unfolding and aggregation^61^.

In our experiments, Tween concentrations in the range of 0.1–3% (v/v) were tested (Fig. 4C). Similar to the results obtained with PEG, LDH activity increased with increasing excipient concentration. The maximum activity achieved for fresh samples was with 0.3% Tween 20, at an activity level of 112 ± 2% (P = 0.04). Further increase in excipient concentration did not change the activity (ANOVA P = 0.77 for all samples containing Tween 20 in the range of 0.3–3%). For desiccated samples, maximum activity was observed in the presence of 1% Tween 20 at a level of 118 ± 10% (P = 0.04), decreasing as the concentration of Tween 20 was increased. Since the main goal was to preserve the biomarker level such that post-desiccation activity is within 5% of that before desiccation (fresh), we selected 0.1% as the optimum concentration with a post-desiccation recovery of 101 ± 4% (P = 0.55). Note that this concentration was considerably lower than that used in HAP studies (Fig. 3) where aggregation of albumin was observed.

Gluconic acid and glucamine (Fig. 4D,E) were used because of their ability to impede moisture-induced aggregation of serum albumin when exposed to high relative humidity in the lyophilized state. The mechanism of protection is thought to be due to the excipient-water interactions, which compete against protein-water interactions^62^.

Concentrations in the range of 0.1–3% (w/v) for both excipients were tested. The presence of gluconic acid had minimal effect on the activity of fresh samples (ANOVA P = 0.33). In desiccated samples, activity increased with increasing gluconic acid concentration, reaching a maximum at 0.3% (98 ± 5%; P = 0.55) followed by reduction in activity with further increase in concentration (down to 87 ± 2% at 3%). Addition of glucamine to the matrix resulted in LDH activity decrease proportional to excipient concentration, with almost complete activity loss at concentrations greater than 0.3%. This behavior was more pronounced in fresh samples. In desiccated samples, LDH activity did not decrease considerably in the range of 0.1–0.3% glucamine. At 0.2% glucamine concentration, maximum LDH activity was achieved with 100 ± 5% recovery (P = 0.91), while further increase in excipient concentration resulted in a significantly reduced activity (with 5.5 ± 0.3% activity remaining at 3% glucamine). This indicated that at low levels, the amine could induce protein stability by hampering aggregation while higher concentrations promoted destabilization.

As gluconic acid, which has a similar structure to glucamine but possesses a carboxyl group instead of an amine, did not exhibit the same tendency to reduce LDH activity, it was inferred that the presence of the amino group in glucamine is destabilizing at high concentrations. This effect was alleviated by desiccation as the desiccated samples, despite longer exposure to glucamine, exhibited a significantly higher activity compared to the fresh controls (e.g. with 0.2% glucamine, desiccated samples had 100 ± 5% recovery, while fresh samples with the same amount of glucamine had 62 ± 0.3% recovery). Gluconic acid and glucamine concentrations of 0.3% and 0.2%, respectively were selected for further testing.

Since we aimed to produce an adsorbent/dissolving lyoprotectant matrix with global protective capabilities, all of the excipients examined here were included at their optimized concentrations to formulate the final lyoprotectant cocktail. Addition of all excipients (Fig. 4F) to the matrix resulted in a considerable increase in fresh sample LDH activity (126 ± 3%). On the other hand, post-rehydration LDH activity in samples isothermally vitrified in the optimized matrix is 104 ± 3% (P = 0.14), indicating that this composition was successful in stabilizing LDH during desiccation.

### Stabilization of Additional Cancer Biomarkers

The optimized matrix was validated using a number of biomarkers including CRP, PSA, MMP-7, and C3a, which exhibit different levels of sensitivity to freezing, desiccation, and storage. For example, CRP and PSA are relatively stable during freeze/thaw, while MMP-7 and C3a are more prone to damage. ELISA assays were used here to determine protein recovery following desiccation and rehydration, because: a) in addition to signaling the presence of the biomarker in the sample, ELISAs are quantitative, providing information on the biomarker level, b) they are used clinically, and c) they are highly specific (as two antibodies are used against a targeted molecule). The use of a monoclonal antibody for detection further enhances the specificity of the assay as it recognizes a single epitope, allowing detection and quantification of small structural changes in the antigen.

**Figure 5:**
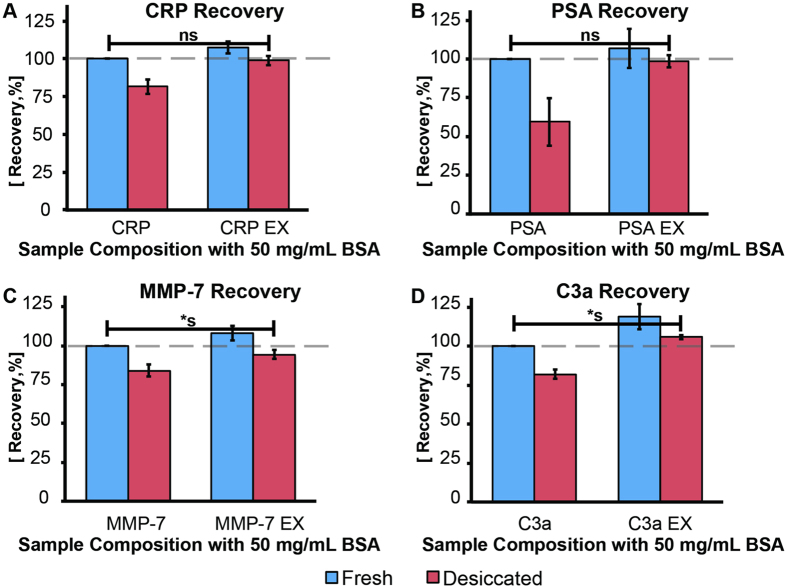
Validation of the optimized matrix by stabilization of biomarkers representing various storage sensitivities. Optimized matrix (EX, red bars) post-rehydration recovery comparison with the fresh, untreated control; highlighted by the connecting bar. (**A**) **CRP:** Complete stabilization (99±3%; P = 0.58) (**B**) **PSA:** Complete stabilization (99±4%; P = 0.47) (**C**) **MMP-7:** Significantly lower recovery post-rehydration (94±3%; P = 0.0015) (**D**) **C3a**: Significantly higher level post-rehydration (106±1%; P = 0.003). Error bars represent standard deviation from at least three independent experiments. Statistically significant difference is denoted by *s (p ≤ 0.05), while non-significant difference is denoted by **ns.**

CRP is a biomarker that is stable against freeze-thaw (up to 7 times), refrigeration, and storage at room temperature for up to 14 days^45^,^46^. When CRP was spiked into the model serum solution containing the matrix excipients (CRP EX, fresh), a moderately elevated value was measured (107 ± 4%), as compared to the untreated control (CRP, fresh) (Fig. 5A). Desiccation of the model serum solution in matrix V1 resulted in 82 ± 5% recovery (CRP, desiccated), while desiccation using the optimized matrix (CRP EX, desiccated) enabled complete stabilization of the protein with 99 ± 3% post-rehydration recovery (P = 0.58).

PSA is a biomarker that is relatively stable during cryogenic and refrigeration storage and against repeated freeze/thaw (up to five times)^48^. Similar to the result obtained with CRP, addition of matrix excipients to the model serum solution spiked with PSA (PSA EX, fresh) resulted in a moderate increase in the measured analyte level (107 ± 13%)(Fig. 5B). Desiccation without excipients resulted in a protein level of 59 ± 16% (PSA, desiccated) as compared to the fresh control. However, isothermal vitrification in the optimized matrix (PSA EX, desiccated) increased post-rehydration recovery to 99 ± 4% (P = 0.47).

MMP-7 is known to be highly sensitive to freeze/thaw^49^. When the model serum solution spiked with MMP-7 was mixed with the lyoprotectant matrix (MMP-7 EX, fresh), the detected protein level exhibited 8 ± 5% increase as compared to the control (Fig. 5C). A level of 84 ± 4% was obtained when the biomarker was desiccated in matrix V1 (MMP-7, desiccated). Post-desiccation recovery increased to 94 ± 3% (P = 0.0015) when the sample was isothermally vitrified using the optimized matrix (MMP-7 EX, desiccated). Even though the difference was small (<6%), post desiccation recovery was statistically significantly different from the fresh control value.

C3a is freeze-stable, but is sensitive to refrigeration and repeated freeze/thaw. More than 50% increase in C3a level after freezing and thawing, compared to fresh controls, have been observed, which is significant enough to obscure analysis and result in false positive diagnoses^26^,^50^. Similar to what was observed in the preceding experiments, addition of the matrix excipients (C3a EX, fresh) elevated the C3a ELISA signal to 119 ± 8% (Fig. 5D). Samples desiccated in matrix V1 (C3a, desiccated) resulted in 82 ± 3% recovery, while post-desiccation level of samples isothermally vitrified in the optimized matrix (C3a EX, desiccated) was 106 ± 1% (P = 0.003). The difference between the fresh and post-desiccation levels were statistically significant but small (<6%) and is deemed to be minimal when compared to the increase of more than 50% observed during freezing and thawing of serum and plasma samples^26^.

The elevated C3a levels in serum and plasma samples subjected to freezing and freeze-thaw, is likely due to the artefactual cleavage of C3 during these processes, producing more C3a^26^. Addition of citrate or EDTA to serum, and plasma samples has been shown to inhibit this effect^50^. Another explanation for the elevated levels observed in this work (also observed in fresh samples treated with the matrix), is matrix interference. This occurs when antibodies used in ELISA non-specifically interact with the matrix resulting in an artificially elevated value. This effect can be mitigated by identifying and reducing the concentration of the interfering component, or by further sample dilution, which would minimize these non-specific interactions.

Isothermal vitrification is not without any downside. One disadvantage of this stabilization method is the requirement for long drying times (4–12 hours at ambient temperature and pressure), which can introduce a certain level of damage, since until the sample is vitrified, the biomolecules are mobile and can undergo processes such as unfolding, degradation, and aggregation. To accelerate desiccation, we employed vacuum drying and achieved >90% water removal within 4 hours resulting in a T_g_ > 4 °C. This allowed storage in a regular refrigerator. To be able to reach room temperature storage, drying times need to be increased.

Another limitation of the isothermal vitrification approach is the need for the removal of the lyoprotectant excipients from the sample after storage if analytical tests susceptible to the presence of carbohydrates in the solution will be utilized. In our experiments, no significant matrix interference was observed when functional assays (e.g. Western Blot, ELISA and enzyme activity measurement) were employed. However, for mass spectrometry, which is routinely used for biomarker discovery^63^, the presence of matrix excipients in the sample may pose a problem. We propose the use dialysis or ultrafiltration to remove low molecular weight excipients, while enzymatic degradation may be considered for the removal of the high molecular weight excipients. Serum and plasma samples are inherently complex fluids. Biochemical, and chromatographic fractionation are often applied to decrease protein complexity and simplify mass spectrometry analyses^63^. Similar processes may be adopted to remove the matrix excipients prior to these analyses.

## Conclusion

In this communication, we report the development and validation of a novel adsorbing/dissolving lyoprotectant matrix to be used for room temperature stabilization and storage of archival human serum specimens by isothermal vitrification. This technology enables biospecimen volume to be reduced to approximately 5% of its initial volume and eases the stringent cryogenic storage/transport requirements, providing significant cost savings. Additionally, this method eliminates the need for thawing, re-aliquoting, and re-freezing of larger specimens (a practice that causes damage accumulation)^11^ since dried samples can easily be broken into smaller pieces for individual assays.

The composition of the lyoprotectant adsorbing/dissolving matrix was optimized to stabilize a wide variety of cancer biomarkers and ensure that post-storage functional clinical assay results match those on the day of collection (with less than 5% error). Here, we demonstrated stabilization of selected cancer biomarkers (LDH, CRP, PSA, MMP-7, and C3a) proving the technical feasibility of isothermal vitrification technology. Research is currently underway to validate the technology for all FDA approved cancer biomarkers and other high value macromolecules in the serum that are currently being evaluated/validated as cancer biomarkers. Note that the technology developed here has the flexibility to be used not only for the preservation of human serum but also other biospecimens such as synovial, cerebrospinal, ascites, and bronchial lavage fluids as well as saliva, sputum, urine, etc. where proteinaceous biomarkers are present.

## Materials and Methods

### Chemicals

Experiments were performed using trehalose dihydrate (≥99% purity, Ferro-Pfanstiehl Laboratories, Waukegan, IL), dextran (35–45 kDa, Sigma-Aldrich, St. Louis, MO), bovine serum albumin (BSA) (≥98% Purity, Sigma), and 1X Dulbecco’s phosphate buffered saline (PBS) solution (14190-144, Invitrogen Corporation, Burlington, Ontario, Canada). All other chemicals (unless indicated) were purchased from Sigma.

### Lyoprotectant cocktail design

The lyoprotectant cocktail was mainly composed of dextran and trehalose (called “high concentration excipients”). In order to further enhance glassy state stability of the biomarkers, five minor excipients (called “low concentration excipients”) namely, glycerol, polyethylene glycol (PEG), Tween 20, gluconic acid, and glucamine were added to the trehalose-dextran cocktail. Experiments conducted to determine the optimum concentrations of the low concentration excipients in the lyoprotectant cocktail are described in detail in the Results and Discussion section.

### Electrospinning

Twenty mL of distilled water that contained low concentration excipients was used to dissolve trehalose and dextran at concentrations of 0.4 g/mL and 1 g/mL, respectively. First, trehalose was dissolved by stirring the solution at 200 RPM for 45 minutes. Dextran was later added in multiple steps and the mixture was allowed to stir overnight (16 hours) at 200 RPM. The solution was stirred at 150 RPM the following day for an additional three hours to eliminate most of the bubbles that formed during mixing. The solution was then allowed to rest for 12 hours to ensure total dissolution. The solution was stored at 4 °C when not in use.

The resultant lyoprotectant cocktail was filled into a 1 mL syringe connected to a stainless steel, 18-gage, 0.5”, long blunt-end needle. The syringe was then placed in a multi-channel syringe-pump (NE-1600 multi-syringe pump; New Era Pump Systems, Farmingdale, NY) and the solution was electrospun at a flowrate of 0.03 mL/min. Electrospinning was conducted in a controlled environment chamber (Electro-tech Systems, Inc., Glenside, PA) with the relative humidity kept constant at 50%. The tip of the needle was kept at a 15 cm distance from an aluminum target, and a voltage differential of 15–20 kV was applied (between the tip of the needle and the target). The voltage applied depended on the number of tips used simultaneously for production (15 kV for 1 tip, 18 kV for 2 tips and 20 kV for 3 tips). These conditions were optimized to result in the most uniform electrospun fiber diameter with optimum inter-fiber distance in the matrix (optimized for capillary adsorption speed vs. dissolution rate), resulting in a well-controlled architecture (Fig. 1C). Every 4 hours, electrospun fibers were collected from the target, and the syringes were refilled with fresh lyoprotectant cocktail. All collected electrospun fibers were sealed and stored in a refrigerator (4 °C), if not used immediately.

### Characterization – *SEM imaging and water content analysis*

Characterization of the electrospun fiber diameter distribution and inter-fiber distance in the matrix were performed by Scanning Electron Microscopy (SEM) imaging. For this purpose, a Hitachi 4700 Field-emission scanning electron microscope (FE-SEM) was used. The specimens for SEM imaging were gold-palladium coated with a VCR ion beam sputter coater at a working distance of 5 mm with an accelerating voltage of 5 kV. To examine the effects of water content on the fiber characteristics and morphology, samples were harvested in triplicate and dried in a vacuum chamber for up to 72 hours. Water content was determined by heating the samples to 130 °C for 60 minutes and calculating the change in the sample weight.

### Characterization – *Glass transition temperature measurement*

The glass transition temperature (T_g_) of the desiccated serum samples adsorbed into the electrospun matrix was measured using a Q1000 (TA Instruments Inc., New Castle, DE) Differential Scanning Calorimeter (DSC). The sample to be tested (weighing 2–10 mg) was loaded into a hermetically sealed aluminum pan. The sample was then rapidly cooled down to −60 °C, equilibrated for 15 minutes, and then warmed up to 150 °C at a constant rate of 2 °C/min. Shift in the DSC scan baseline indicated a glass transition and the corresponding temperature was recorded as T_g_.

### Collection of human serum samples

Human blood samples were collected from volunteers by University of Minnesota Tissue Procurement Facility (TPF) staff following a University of Minnesota Institutional Review Board (IRB) approved protocol (Study Number: 1011E92892). Informed consent was obtained by TPF from all volunteer subjects. The experimental methods were approved by University of Minnesota IRB, and experiments were carried out in accordance with the approved guidelines.

Whole blood was processed to separate serum by allowing it to clot for approximately 20–30 minutes after receipt of the samples from TPF. Each vial of whole blood produced approximately 40% the blood volume as sera. The clotted blood was then centrifuged for 10 min at 2000 RCF. The serum (the supernatant) was then carefully aspirated at room temperature and placed into a new centrifuge tube, taking care not to disturb the cell layer or transfer any cells. Serum samples were then aliquoted into microcentrifuge tubes and allocated to different experimental groups as detailed in Table 1.

In desiccation experiments, 150 μL of serum was adsorbed into 250 mg of electrospun matrix. The samples were then either sealed, allowed to diffusively dry in a desiccation chamber at 50% RH, or vacuum dried for 4 hours. Rehydration of the isothermally vitrified samples was done by adding 100 μL of ultrapure water (to make up for the water lost during drying for 4 hours) and 1.35 mL of 1X PBS to make a 1:10 dilution of the original sample and stirring for 1 hour. Dilution was performed in order to facilitate re-suspension of the isothermally vitrified samples. This did not influence the results since ultra low concentrations (picogram to microgram per milliliter) of protein were required for the following experiments (SDS-PAGE, ELISA, Western Blot, and LDH assay) where samples were diluted 500–5000 times prior to analysis. A similar process was followed for model serum samples used in the experiments to finalize the lyoprotectant matrix composition.

### Characterization – *Determination of Protein Concentration*

Before any analysis, total protein content of the specimen was determined using BCA protein assay following the protocol provided by the supplier (Pierce™ BCA Protein Assay Kit, ThermoFisher Scientific, Waltham, MA).

#### SDS-PAGE

Sodium dodecyl sulfate polyacrylamide gel electrophoresis (SDS-PAGE) was conducted to examine degradation, aggregation, or depletion following isothermal vitrification, storage, and rehydration. Details of the process can be found elsewhere^64^. Briefly, 1 μg serum protein from each sample was denatured in loading buffer (containing 0.625 M Tris, 10% (w/v) glycerol, 0.05% bromophenol blue, 1% SDS, and 1% mercaptoethanol) and separated on a precast 4–20% gradient gel (BioRad, Hercules, CA) in Tris-glycine buffer (25 mM Tris, 192 mM glycine) with 0.1% SDS.

#### Silver stain

In order to visualize changes in protein structure (e.g. aggregation and degradation) following treatment, silver staining was used following the protocol provided in the Bio-Rad Silver Stain Plus Kit.

#### Western blots

Western blot analysis was used for detection of the high abundance serum proteins, albumin and haptoglobin^64^. Briefly, protein transfer to polyvinylidene difluoride (PVDF) membranes (GE Healthcare Biosciences, Pittsburgh, PA) following gel electrophoresis was conducted in Tris-glycine buffer containing 12.5% (v/v) methanol. Five percent nonfat dry milk and 0.05% Tween-20 in PBS was then used to block the PVDF membranes overnight. The membranes were then incubated in a rabbit anti-human haptoglobin antibody (1:20,000 dilution, ab85846, Abcam, Cambridge, MA) or rabbit anti-human serum albumin (Advanced Targeting Systems, San Diego, CA) in the blocking solution. The membranes were then washed three times with PBS that contained 0.05% Tween-20 and incubated in goat anti-rabbit horseradish peroxidase-labeled secondary antibody (1:10,000 dilution, 1858415, Pierce) in the blocking solution. West Femto chemiluminescent substrate (Pierce) was used to for detection and Kodak x500 film (Midwest Scientific, Valley Park, MO) was used for imaging.

#### Enzyme activity assay

An enzymatic activity assay was conducted to monitor the stability of lactate dehydrogenase (LDH) during matrix optimization, following the protocol provided by the supplier (ab102526, Abcam, Cambridge, MA).

#### ELISA

ELISA testing was conducted to measure the recovery of the selected cancer biomarkers in isothermally vitrified samples following rehydration. Sandwich ELISA kits for CRP (RAB0096, Sigma, St. Louis, MO), PSA (ab188388, Abcam, Cambridge, MA), MMP-7 (RAB0369, Sigma, St. Louis, MO), and C3a (BMS2089, affymetrix eBioscience, San Diego, CA) were used in these experiments.

### Statistical Analysis

All values for protein activity/recovery for treated samples (fresh samples that contained selected excipients or desiccated samples with/without excipients) were compared individually to the values obtained for the untreated (fresh) control (taken as 100%). Statistical significance was determined using a two-tailed t-test, and the results reported were the mean of at least 3 replicates ± standard deviation (SD). To determine variability in biomarker activity/recovery in response to different conditions (i.e. excipient composition or concentration), a one-way ANOVA was conducted. P-values ≤ 0.05 were considered statistically significant. Note that the P-values were obtained by comparing the experimental group with the untreated (fresh) control, unless indicated otherwise.

## Additional Information

**How to cite this article**: Solivio, M. J. *et al.* Adsorbing/dissolving Lyoprotectant Matrix Technology for Non-cryogenic Storage of Archival Human Sera. *Sci. Rep.* **6**, 24186; doi: 10.1038/srep24186 (2016).

## Supplementary Material

Supplementary Information

## Figures and Tables

**Table 1:** Experiments Conducted to Determine High Abundance Protein (HAP) Stability in Desiccated, Frozen, or Frozen/Thawed Human Serum.

Experiments Run	Preservation Protocol	Storage Conditions/Time	Methods used for Stability Evaluation ⇒Conclusion
Human Serum (n = 3)	IV* in V1, V2, and V3 (vs. fresh control)	At 22 °C & 0%RH for 1, 3, 7, 16, 18, 20, 22 days	Silver Stain, Western Blot for Albumin and Haptoglobin ⇒ HAP aggregation in dried samples that were sealed
Human Serum (n = 3)	IV in V2, V3 vs. freezing at −20 °C or −80 °C (vs. fresh control)	At 22 °C & 0%RH for 1, 3, 7 days	Silver Stain, Western Blot for Albumin and Haptoglobin ⇒ HAP aggregation in dried samples that were sealed, degradation in frozen samples
Human Serum (n = 2)	IV in V3 vs. freezing at −20 °C or −80 °C (vs. fresh control)	At 4 °C or 22 °C & 0%RH for 1, 3, 7 days with/without UV exposure	Silver Stain, Western Blot for Albumin and Haptoglobin ⇒ Significant decrease in HAP aggregation ⇒ Liquid sera samples stored in cryogenic conditions have high molecular weight aggregates
Human Serum (n = 2)	IV in V2, V3 vs. 4 °C, freezing at −20 °C or −80 °C, 5 Freeze/Thaw to −80 °C (vs. fresh control)	At 4 °C or 22 °C & 0%RH for 1, 2, 4, 6 weeks with/without UV exposure	Silver Stain, Western Blot for Albumin and Haptoglobin ⇒ Frozen state storage causes aggregation/degradation equal to or greater than stabilization matrix ⇒ Significant aggregation during freeze/thaw ⇒ An interesting high molecular weight band which appears predominantly in samples stored at 4 °C
Human Serum (n = 3)	IV in V3 vs. freezing at −20 °C or −80 °C, 5 Freeze/Thaw to −80 °C (vs. fresh control)	At 4 °C or 22 °C & 50%RH for 1 week	Silver Stain, Western Blot for Albumin and Haptoglobin ⇒ Degradation in 50%RH samples ⇒ Serum dried w/o lyoprotectant cocktail aggregated the worst
Human Serum (n = 3)	IV in V2, V3 vs. freezing at −20 °C or −80 °C	At 4 °C or 22 °C for 2 years with/without UV exposure	Silver Stain, Western Blot for Albumin and Haptoglobin ⇒ Experiments are ongoing

^*^Isothermal vitrification (IV) matrices used in experiments:*V1*: Trehalose + Dextran; *V2*: Trehalose + Dextran + Tween 20; *V3*: Trehalose + Dextran + Tween 20 + Glycerol.
