# Supplementary material for: Adsorbing/dissolving Lyoprotectant Matrix Technology for Non-cryogenic Storage of Archival Human Sera
Source: Sci Rep. 2016 Apr 12;6:24186. doi: 10.1038/srep24186 (PMC4828708; doi:10.1038/srep24186)

Adsorbing/dissolving Lyoprotectant Matrix Technology for Non-cryogenic Storage of Archival Human Sera

Morwena J. Solivio[1], Rebekah Less[1, 2], Mathew L. Rynes[1], Marcus Kramer[1], Alptekin Aksan[1*]

[1]*Biostabilization Laboratory, Department of Mechanical Engineering, University of Minnesota, Minneapolis, MN 55455, USA*

[2]*School of Biomedical Engineering and Sciences, Virginia Tech, Blacksburg, VA 24061, USA*

Supporting Information

Table of Contents

		Page
**Figure S1:**	**Silver stained gel showing sample profiles in various storage conditions**	**S-2**
**Figure S2:**	**Effect of repeated freezing and thawing on LDH activity**	**S-3**

Figure S1. Silver stained gel showing sample profiles in various storage conditions. Lanes 1 and 2 contain serum samples initially stored under

cryogenic conditions, -80°C and -20°C. While lanes 3-6 contain samples that were desiccated using the optimized matrix, stored for 1 or 3 days. Lanes 5-6 contain samples vitrified in matrix containing 0.5% Tween 20. A different profile can be observed for frozen samples, with high molecular weight aggregates being distinctly observed. No high molecular weight aggregates were observed for desiccated samples with and without Tween 20. This figure is in support for **Figure 3** in the manuscript, showing that in unsealed samples, Tween 20 alone does not cause aggregation.

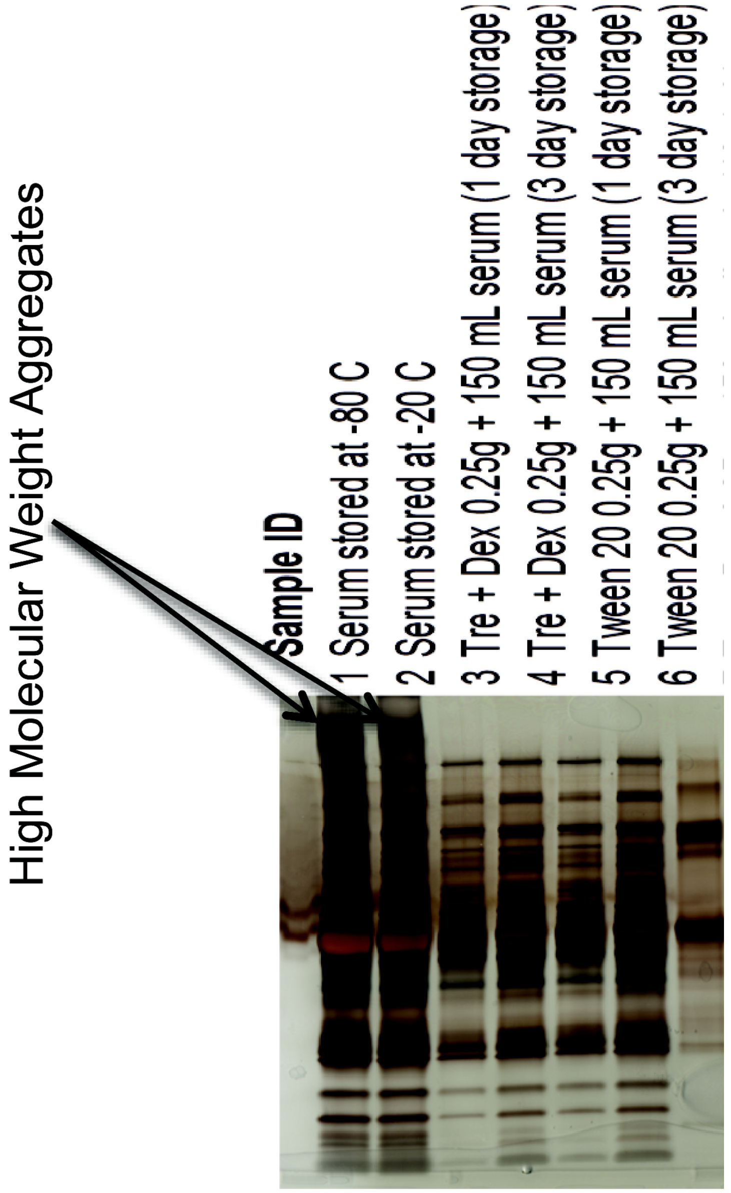

Figure S2. Effect of repeated freezing and thawing on LDH activity. LDH solutions containing different concentrations of BSA were subjected to repeated

freezing and thawing to establish the effect of this process on enzymatic activity. In the absence of BSA, LDH loses 51± 5%, 78±1%, and 79±1% of its activity after one, five, and ten cycles, respectively, which has been attributed to the dissociation of the enzyme multimer. In the presence of 5 mg/mL BSA, LDH exhibited maximum stability with 3±1% change in activity after ten cycles. Samples with 50 mg/mL BSA, representing serum conditions exhibited enzymatic activity increase proportional to the number of freeze-thaw cycles with 21±1% and 46±9% change after five and ten freeze-thaw cycles, respectively (n=3). This effect has been attributed to the formation of the more active tetramer from the less active dimer (which is more abundant in serum conditions), due to crowding in the presence of BSA during freezing. This figure is in support for **Figures 4B, 4C and 4F** in the manuscript, where a significant rise in LDH activity is observed in the presence of excipients PEG, Tween 20 and all five excipients, similarly attributed to crowding, which promotes the formation of the more active tetrameric enzyme.

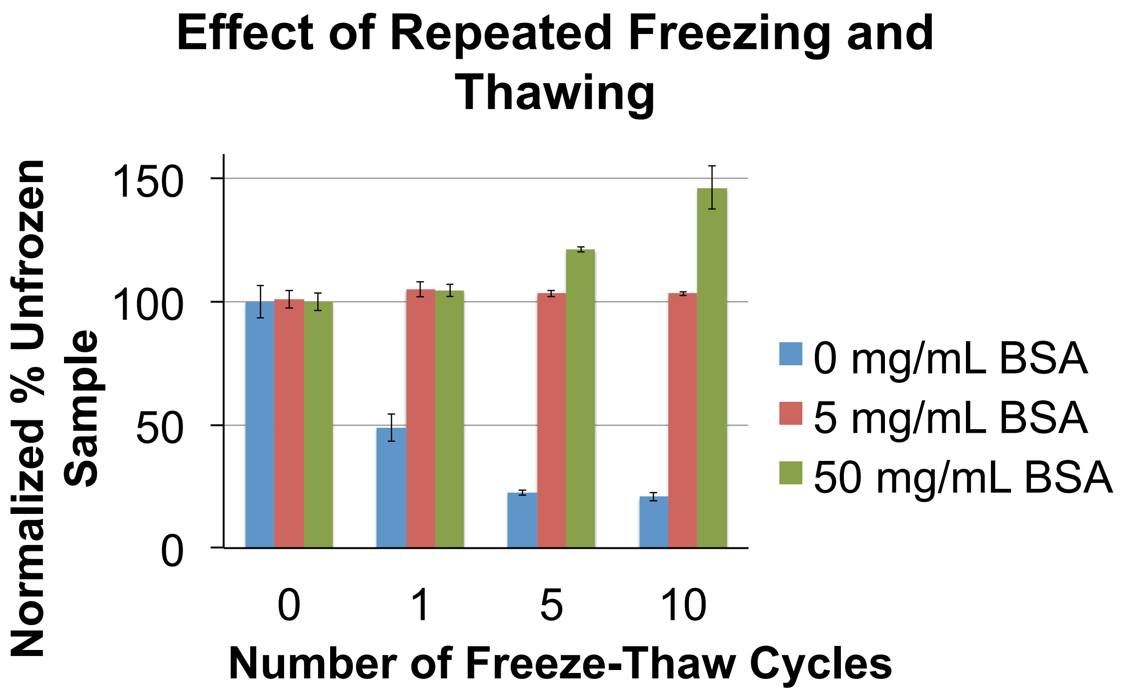

Supplement: Supplementary Information [file srep24186-s1.pdf]
